# Effects of Nandrolone Decanoate on Muscle Strength, Body Composition and Bone Density: A Systematic Review and Meta‐Analysis

**DOI:** 10.1002/jcsm.70276

**Published:** 2026-04-05

**Authors:** Konstantinos Prokopidis, Theocharis Ispoglou, Trevor Thompson, Dolores Sanchez‐Rodriguez, Anna Marie Hergelegiu, Cafer Balci, Mariana Alves, Damiano Pizzol, Joseph McLean, Pinar Soysal, Brigid Unim, Antonio Cherubini, Nicola Veronese, Special Interest Group in Systematic Reviews and Meta‐analyses of the European Geriatric Medicine Society (EuGMS)

**Affiliations:** ^1^ Department of Musculoskeletal Biology, Institute of Life Course and Medical Sciences University of Liverpool Liverpool UK; ^2^ Carnegie School of Sport Leeds Beckett University Leeds UK; ^3^ Centre for Chronic Illness and Ageing University of Greenwich London UK; ^4^ Geriatrics Department Brugmann University Hospital, Université Libre de Bruxelles Brussels Belgium; ^5^ Rehabilitation Research Group Hospital del Mar Research Institute Barcelona Spain; ^6^ Department of Internal Medicine, Faculty of Medicine Carol Davila University of Medicine and Pharmacy Bucharest Romania; ^7^ Ana Aslan National Institute of Gerontology and Geriatrics Bucharest Romania; ^8^ Division of Geriatric Medicine, Faculty of Medicine Hacettepe University Ankara Türkiye; ^9^ Laboratory of Clinical Pharmacology and Therapeutics, Faculdade de Medicina Universidade de Lisboa Lisbon Portugal; ^10^ Serviço de Medicina ULS Santa Maria (ULSSM) Lisbon Portugal; ^11^ Health Unit Eni Maputo Mozambique; ^12^ Androlabs London UK; ^13^ Department of Geriatric Medicine, Faculty of Medicine Bezmialem Vakif University Istanbul Türkiye; ^14^ Department of Cardiovascular, Endocrine‐Metabolic Diseases and Aging National Institute of Health Rome Italy; ^15^ Geriatria, Accettazione geriatrica e Centro di Ricerca per l'Invecchiamento, IRCCS INRCA Ancona Italy; ^16^ Department of Clinical and Molecular Sciences Università Politecnica Delle Marche Ancona Italy; ^17^ Geriatric Unit, Department of Medicine University of Palermo Palermo Italy; ^18^ Saint Camillus International University of Health Sciences Rome Italy

**Keywords:** body composition, bone density, muscle strength, nandrolone decanoate, physical performance

## Abstract

**Background:**

Despite ongoing interest in anabolic therapies for sarcopenia and cachexia, pharmacological interventions are being developed to counteract age‐ and condition‐related losses of muscle mass and strength. This systematic review and meta‐analysis evaluated the effect of nandrolone decanoate on lean soft tissue (LST), fat mass, handgrip strength, knee extension strength and bone mineral density (BMD) in adults.

**Methods:**

Following PRISMA guidelines, we searched PubMed, Scopus, Web of Science and Cochrane Library (inception to April 2025) for randomised controlled trials (RCTs) comparing nandrolone decanoate to placebo in adults ≥ 18 years. Data were analysed using a random‐effects meta‐analysis, and risk of bias was assessed using the RoB2 tool. Mean differences (MD) alongside 95% confidence intervals (95% CI) were applied, whereas standardised MD (SMD) was used when methods or/and units of measurement were not identical (i.e., body fat mass/percentage).

**Results:**

Twenty RCTs were included in this study. Nandrolone decanoate significantly increased LST (k = 11; MD: 1.59 kg, 95% CI 1.06–2.13, *p* < 0.01, *I* ^2^ = 0%) but had no effect on fat mass (k = 11; SMD: −0.04, *p* = 0.65), handgrip strength (k = 12; SMD: 0.39, *p* = 0.10) or knee extension strength (k = 1; *p* = 0.99). BMD outcomes were inconsistent, with only total proximal femur BMD showing improvement (*p* < 0.05). No publication bias was detected. Most studies showed a low risk of bias, whereas GRADE assessment displayed a low certainty of evidence.

**Conclusion:**

Although nandrolone decanoate modestly increases LST, this was not accompanied by improvements in handgrip strength or consistently observed improvements in BMD, and functional outcomes were informed by limited trial data. Taken together, these findings do not support routine clinical use of nandrolone decanoate for improving musculoskeletal health. Given its limited benefit and known safety concerns, non‐pharmacological approaches such as resistance training and nutritional support should remain the preferred strategy for preserving musculoskeletal health.

## 1 Introduction

The musculoskeletal system plays a critical role in human health. The loss of bone mass, muscle mass, muscle function and physical performance due to ageing or concomitant diseases is highly prevalent and has been shown to be strongly associated with adverse outcomes, such as increased disability [1], higher mortality risk [2] and impaired quality of life.

This decline in musculoskeletal health is, at least partly, reversible through targeted interventions, with non‐pharmacological strategies remaining the cornerstone of prevention and treatment [3, 4, 5, 6]. A well‐balanced diet with adequate protein intake [7], regular physical activity [8] and structured resistance exercise [5, 9, 10] are among the most effective and safest approaches for preserving and improving muscle and bone health [6, 8]. Despite these well‐established strategies, in some clinical conditions where muscle wasting or bone loss is severe, pharmacological interventions have also been explored [4]. However, their roles remain secondary, given the risks and limitations associated with their use [3, 5].

Androgenic–anabolic steroids, including testosterone and its derivatives, have been investigated for their potential to enhance muscle protein synthesis and satellite cell proliferation [11], maintain physical function [12] and promote musculoskeletal health [13, 14]. One such compound is nandrolone decanoate, which has been prescribed in specific clinical settings [11, 15]. Nandrolone decanoate has been used in the management of certain conditions characterised by muscle wasting or bone loss, including chronic kidney disease, osteoporosis in postmenopausal women, inoperable breast cancer, HIV‐associated wasting syndrome and other catabolic states caused by chronic diseases or acute conditions, such as acute stress or trauma. Nevertheless, its therapeutic application remains poorly defined, and it has been linked to clinically relevant adverse effects, including infertility, impaired lipid profiles, high blood pressure, acne, hair loss and gynaecomastia. Clinically significant hepatotoxicity is uncommon with injectable nandrolone decanoate at therapeutic doses, with hepatic adverse effects primarily reported following prolonged exposure or supraphysiological use. Robust evidence on both efficacy and safety is therefore required before considering wider clinical use.

Importantly, the non‐medical use of nandrolone decanoate is prohibited in competitive sport under World Anti‐Doping Agency regulations, although its legal status outside sport varies by jurisdiction and regulatory context, and may be associated with significant health risks [16]. Although some professional athletes, including in bodybuilding and weightlifting, are sometimes users of nandrolone decanoate [15], their misuse is strongly discouraged due to the potential for serious adverse effects and ethical concerns related to sports doping [17]. Given these risks, the role of nandrolone decanoate should be carefully evaluated within a strict clinical framework rather than for performance enhancement in healthy individuals.

Despite its use in both clinical and non‐clinical settings, no systematic review or meta‐analysis has evaluated whether reported anabolic effects of nandrolone decanoate translate into clinically meaningful improvements in musculoskeletal and bone health, that is, bone mineral density (BMD), body composition (e.g., muscle mass and fat mass) and physical function (i.e., muscle strength and physical performance). Furthermore, the optimal dose and duration of administration to provide clinically meaningful or significant improvements on musculoskeletal health are also unclear, as is the possibility of sex‐ or age‐dependent responses.

The primary objective of this study was to assess the efficacy of nandrolone decanoate on musculoskeletal and bone health outcomes (i.e., physical function, body composition and BMD) by examining randomised controlled trials (RCTs) in adults. The secondary objectives were to determine (1) the optimal dose and duration of administration to provide meaningful and/or significant improvements and (2) whether age and sex influence the response to nandrolone decanoate.

## 2 Methods

This systematic review and meta‐analysis was conducted in accordance with the Preferred Reporting Items for Systematic Reviews and Meta‐Analyses (PRISMA) guidelines [18]. The protocol was registered in the International Prospective Register of Systematic Reviews (PROSPERO) (registration number CRD42022367273).

### 2.1 Search Strategy

Six independent reviewers (K.P., A.M.H., C.B., M.A., B.U. and L.P.) searched PubMed, Scopus, Web of Science and Cochrane Library from inception until April 2025 using the search strategy outlined in Table 1. Data extraction was performed by four independent reviewers (K.P., T.T., A.M.H. and C.B.). Discrepancies in the literature search process were resolved by other investigators through Covidence software.

**TABLE 1:** Study and participant characteristics of the included studies. Data are expressed as mean (standard deviation).

Study, year	Study design	Health status	Total n (M/F)	Age	Body composition assessment tool	Total treatment dosage (mg)	Treatment duration (weeks)	Controlled dietary intake?
Schols et al., 1995	Double‐blind placebo‐controlled trial	COPD	—	—	BIA	Men: 200; Women: 100	8	Yes
Van Loan (Low dose) et al., 1999	Double‐blind placebo‐controlled trial	HIV	—	36 ± 7.4	BIA	133	2	Yes
Van Loan (High dose) et al., 1999	Double‐blind placebo‐controlled trial	HIV	—	42.4 ± 8.7	BIA	392	2	Yes
Johansen et al., 1999	Double‐blind placebo‐controlled trial	Haemodialysis/peritoneal dialysis	23/6	44 ± 15	DXA	2600	26	Yes
Storer et al., 2005	Double‐blind placebo‐controlled trial	HIV	—	43.9 ± 8.8	DXA	900	12	Yes
Frisoli Jr. et al., 2005	Double‐blind placebo‐controlled trial	Osteoporosis	0/65	74 ± 3.8	DXA	1750	104	No
Johansen et al., 2006	Double‐blind placebo‐controlled trial	Haemodialysis	24/15	55.7 ± 13.4	DXA	Men: 2400; Women: 1200	12	No
Fahey et al., 1973	Double‐blind placebo‐controlled trial	Healthy	28/0	— (College students)	Underwater densitometry	225	9	No
Sloan et al., 1992	Double‐blind placebo‐controlled trial	Hip fracture	—	83 ± 7	BIA	448	4	No
Sharma et al., 2008	Double‐blind placebo‐controlled trial	COPD	9/7	71 ± 10.18	BIA	Men: 400; Women: 200	16	Yes
Loevoy et al., 1996	Randomised, placebo‐controlled trial	Obesity	0/20	— (Postmenopausal)	DXA	570	39	No
Crawford et al., 2003	Double‐blind placebo‐controlled trial	Long‐term glucocorticoid therapy	33/0	62.7 ± 4.2	DXA	2600	26	No
Gold et al., 2006	Double‐blind placebo‐controlled trial	HIV	237/0	41.7 ± 8.8	BIA	1500	12	No
Mulligan et al., 2005	Double‐blind placebo‐controlled trial	HIV	0/38	36^a^	BIA	600	12	Yes
Lichtenbelt et al., 2004	Double‐blind placebo‐controlled trial	Bodybuilders	16/0	31.9 (20.3)	DXA	1600	8	No
Anstey et al., 2022	Double‐blind placebo‐controlled trial	Critically ill	13/9	69.7 (9.6)	—	Men: 600; Women: 300	3	Yes
Daga et al., 2014	Double‐blind placebo‐controlled trial	COPD	29	60.1 (12.2)	—	448	4	No
Creutzberg et al., 2003	Double‐blind placebo‐controlled trial	COPD	63/0	66 (8)	—	200	8	No
Johansen et al., 1989	Double‐blind placebo‐controlled trial	Osteoporosis	0/39	65.8 (5.8)	Single‐photon absorptiometry	850	52	No
Passeri et al., 1993	Double‐blind placebo‐controlled trial	Osteoporosis	0/46	56.3 (4.4)	Single‐photon absorptiometry	850	52	No
Flicker et al., 1997	Double‐blind placebo‐controlled trial	Osteoporosis	0/60	70.6 (8.2)	DXA and DEQCT	1300	104	No

Abbreviations: BIA, bioelectrical impedance; BMI, body mass index; COPD, chronic obstructive pulmonary disease; DEQCT, dual‐energy quantitative computed tomography; DXA, dual x‐ray absorptiometry; HIV, human immunodeficiency virus.

^a^ Represents median age.

### 2.2 Inclusion and Exclusion Criteria

Studies were included based on the following criteria: (i) RCTs with at least one arm using placebo and one using nandrolone and (ii) participants aged ≥ 18 years old. Studies were excluded if they were (i) open‐label studies without a control group, (ii) interventions including additional pharmacological means known to improve physical fitness (i.e., use of other anabolic androgenic steroids) and (iii) reviews, letters, animal experiments, commentaries or studies not published as full text.

### 2.3 Endpoints

Lean soft tissue (LST) (kg), fat mass (kg) and handgrip strength (kg), knee extension (kg) and BMD of the lumbar and radial spine, femoral neck, total proximal femur, total forearm and total body (g/cm^2^ or % change from baseline) were the primary endpoints used in the meta‐analysis. In line with our PROSPERO registration, ‘muscle mass’ was operationalised using LST mass or fat‐free mass, as these were the only muscle‐related body composition measures consistently reported across eligible placebo‐controlled RCTs. Where possible, all data were converted to the same units in order to obtain mean differences (MDs) between nandrolone and placebo groups for both primary and secondary outcomes.

### 2.4 Risk of Bias and Quality of Evidence

The quality of included studies was evaluated using the risk‐of‐bias 2 (RoB2) tool [19] by three independent reviewers (K.P., J.M. and B.U.). Discrepancies were resolved by another member of the team (N.V.). RoB2 is a comprehensive tool used to assess bias in RCTs across the following domains: (i) randomisation process; (ii) deviations from intended interventions; (iii) missing outcome data; (iv) measurement of the outcome; and (v) selection of the reported result. According to the scoring system, study bias was defined as ‘high’, ‘some concerns’ or ‘low’ [20].

The GRADE approach was used to assess the quality of evidence for each outcome from the meta‐analysis. Factors evaluated included risk of bias, inconsistency, indirectness, imprecision and publication bias. Evidence quality was rated as high, moderate, low or very low.

### 2.5 Statistical Analysis

Our meta‐analysis compared the impact of nandrolone decanoate versus placebo on LST, fat mass and handgrip strength. Quantitative data were treated as continuous measurements, and changes in outcomes from baseline to follow‐up were compared between groups to calculate effect sizes. For LST, effect size was the MD in pre–post weight gain between nandrolone and control groups measured in kg. For fat mass and change in grip strength, we used the standardised mean difference (SMD), given that different studies assessed these outcome with different metrics, utilising either pre–post change in kg or percentage change from baseline.

Meta‐analysis was conducted using a random‐effects model, given likely heterogeneity of effect sizes due to variation in study characteristics. Estimation was conducted using the Hunter–Schmidt estimation method with a small sample‐size correction [21]. The classification of data as moderately inconsistent was based on Higgins' *I* ^2^ from 50% to 74.9% and highly inconsistent from 75% and above.

Furthermore, publication bias was assessed by visually inspecting funnel plots and using the Egger's bias test. To explore sources of heterogeneity, we conducted multiple random‐effects meta‐regressions [22], examining the tool used for the assessment of body composition, whether studies assessed and accounted for dietary intake, participant age and treatment duration. We explored sex as a potential effect modifier using study‐level female proportion in meta‐regression. This analysis was exploratory and does not permit sex‐specific inference. In relation to the systematic review, a narrative synthesis was used [23].

## 3 Results

### 3.1 Search Results

**FIGURE 1:**
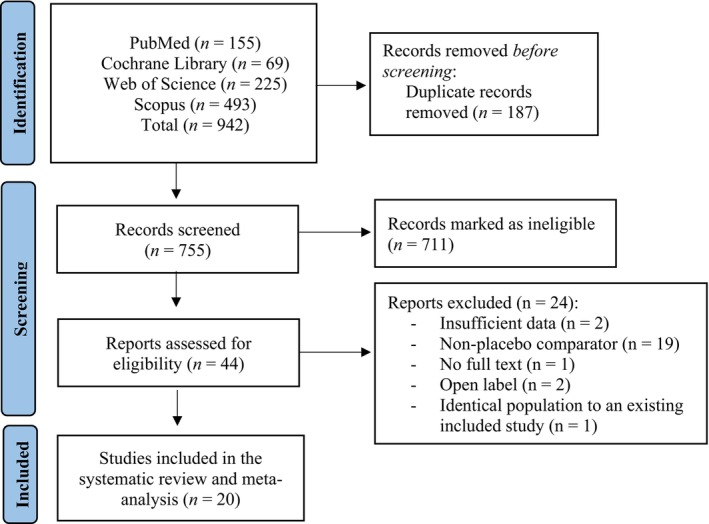
Flowchart of the search strategy employed in the literature search.

Our search yielded 942 articles, of which 187 were removed as duplicates. Out of 755 screened records, 711 were excluded based on title and abstract, and of the remaining 44, 19 had a non‐placebo comparator [24, 25, 26, 27, 28, 29, 30, 31, 32, 33, 34, 35, 36, 37, 38, 39, 40, 41, 42], two had insufficient data [43, 44], one was open label [45], one had no full text [46], and one had a portion of participants identical to ones included in another trial [47] in this meta‐analysis. In total, 20 studies were included in the systematic review and meta‐analysis (Figure 1) [15, 48, 49, 50, 51, 52, 53, 54, 55, 56, 57, 58, 59, 60, 61, 62, 63, 64, 65, 66]. Of these, one study included college students without reporting age specifics, but the highest mean age across studies was 83 years. Six studies used bioelectrical impedance (BIA), eight used dual x‐ray absorptiometry (DXA; from which one used also dual‐ energy quantitative computed tomography), one used underwater densitometry, and two used single‐photon absorptiometry. Treatment duration ranged from 2 to 104 weeks, and based on available data, 37.9% of participants were females. Finally, seven studies controlled for dietary intake. Detailed characteristics of the studies are presented in Table 1.

### 3.2 Effect of Nandrolone Decanoate on LST, Fat Mass and Handgrip Strength

**FIGURE 2:**
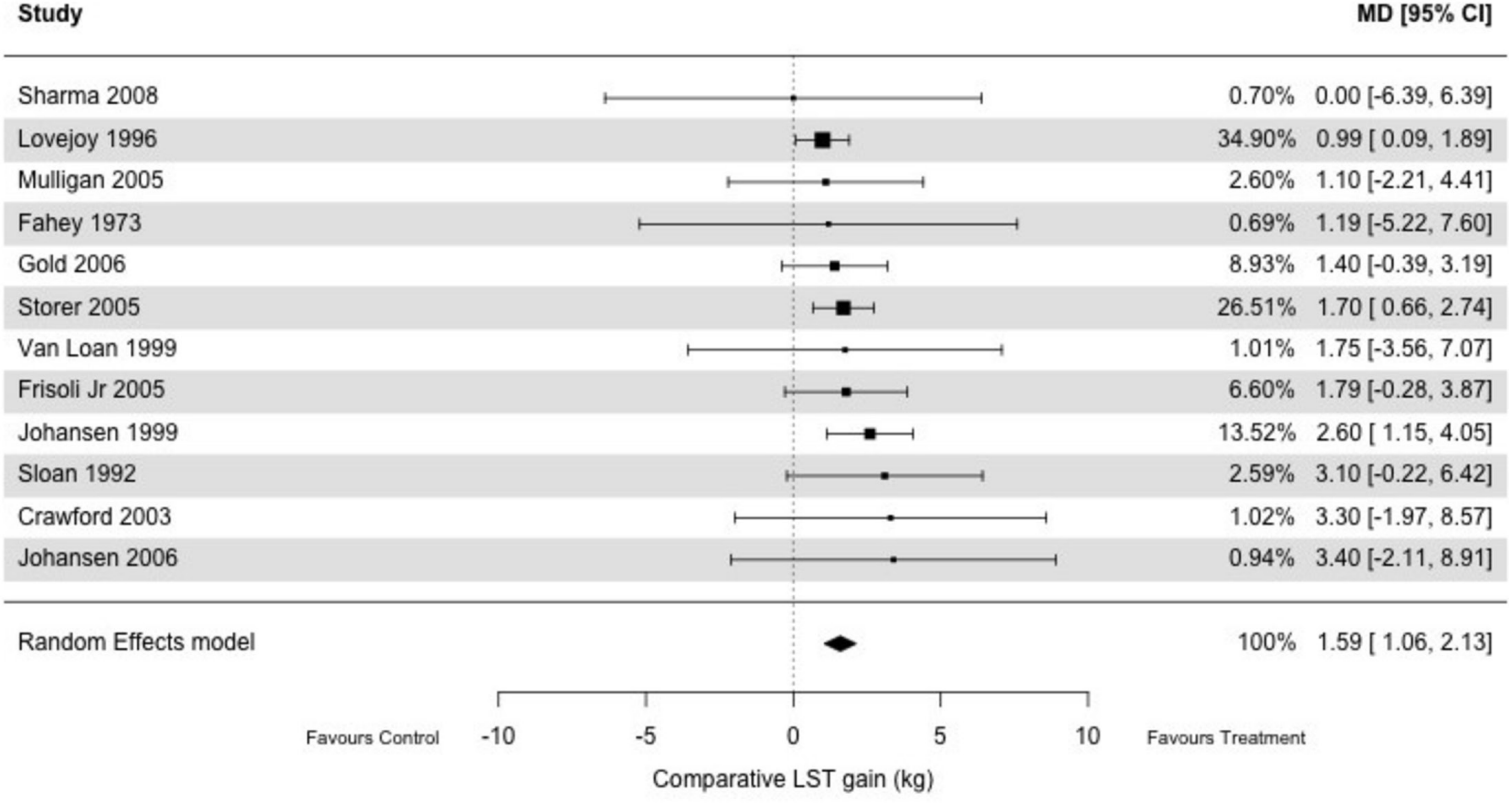
Effect of nandrolone decanoate versus placebo on lean soft tissue.

**FIGURE 3:**
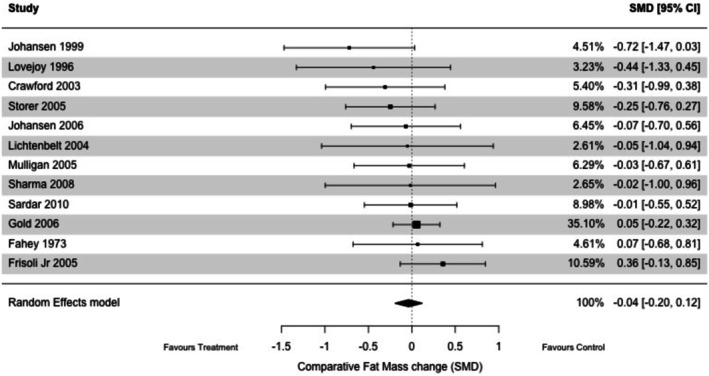
Effect of nandrolone decanoate versus placebo on fat mass.

**FIGURE 4:**
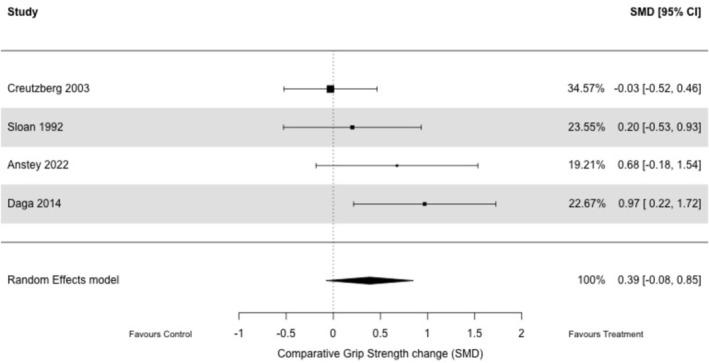
Effect of nandrolone decanoate versus placebo on handgrip strength.

Supplementation with nandrolone decanoate versus placebo led to a statistically significant increase in LST (used as a proxy for muscle mass) (*k* = 12; nandrolone *n* = 358 vs. placebo *n* = 259; MD: 1.59 kg, 95% CI 1.06–2.13, *p* < 0.01, *I* ^2^ = 0%) (Figure 2). No changes were observed in terms of fat mass (*k* = 12; nandrolone *n* = 383 vs. placebo *n* = 265; SMD: −0.04, 95% CI: −0.20–0.12, *p* = 0.65, *I* ^2^ = 0%) (Figure 3) and handgrip strength (*k* = 4; nandrolone *n* = 79 vs. placebo *n* = 67; SMD: 0.39, 95% CI −0.08–0.85, *p* = 0.10, *I* ^2^ = 44%) (Figure 4).

No publication bias was found for any outcome of interest (Table S2). Likewise, we found no covariates that significantly modified the results of our analysis based on meta‐regressions, including the exploratory moderator analysis using study‐level female proportion (Table S3).

### 3.3 Effect of Nandrolone Decanoate on Knee Extension Strength

Only one study assessed the impact of nandrolone decanoate on knee extension strength through three repetition maximum (3RM) [58], precluding any pooled analysis or firm inference. No changes were observed after 12 weeks (nandrolone change: 1.4 [2.1] lb.; placebo change: 0.8 [2.0] lb., *p* = 0.99).

### 3.4 Effect of Nandrolone Decanoate on BMD

Spine BMD remained unchanged in one study after 52 weeks (nandrolone change: 0.002 [0.080] g/cm^2^ placebo change: −0.010 [0.064] g/cm^2^) (*p* > 0.05) [56]. Radial BMD also remained unchanged (nandrolone change: 2.07 [1.22]%; placebo change: −2.57 [1.17]%) (*p* > 0.05) [61]. In another study [53], 104 weeks of nandrolone decanoate supplementation did not increase lumbar spine BMD (nandrolone change: 3.0 [2.2]%; placebo change: 1.6 [1.3]%, *p* > 0.05) nor femoral neck BMD (nandrolone change: 1.9 [1.9]%; placebo change: −0.1 [1.9]%, *p* > 0.05) or total forearm BMD (nandrolone change: −0.4 [0.9]%; placebo change: −1.8 [1.2]%), but increased total proximal femur BMD (nandrolone change: 3.8 [1.8]%; placebo change: 0.7 [1.1]%, *p* < 0.05), using DXA. Additionally, no effect was observed when dual‐energy quantitative computed tomography (DEQCT) was used to measure lumbar spine BMD (nandrolone change: 11.2 [6.0]%; placebo change: 7.8 [4.1]%, *p* > 0.05). Finally, in another RCT [15], no changes were demonstrated after 14 weeks on total BMD (nandrolone decanoate pre: 1.279 [0.091] g/cm^2^–nandrolone decanoate post: 1.274 [0.075] g/cm^2^; placebo pre: 1.328 [0.092] g/cm^2^–placebo post: 1.338 [0.086] g/cm^2^) (*p* > 0.05). Overall, BMD findings were derived from a small number of heterogeneous studies using different skeletal sites and assessment methods, precluding pooling and limiting interpretability.

Reported adverse events are presented in the Supporting Information.

### 3.5 Risk of Bias and GRADE Approach

Of the included studies, six were judged to have some concerns overall [48, 51, 52, 57, 61, 64], whereas the rest had a low overall risk of bias. Three studies had at least one domain as high risk of bias [52, 61, 63], due to a lack of baseline detail and statistical differences between the nandrolone and the placebo group at baseline. One study had some concerns due to lack of blinding [53]. In other studies, there was no information on allocation concealment [15, 51, 56, 59, 64], or the randomisation process was not described [60]. A notable age difference between groups was observed in two studies [48, 57]. Finally, one study had some concerns due to missing data and high dropout rates [65]. Risk of bias assessment is shown in Table S4.

GRADE assessment (Table S5) displayed low certainty pertaining to results around fat mass, LST and handgrip strength changes, moderate certainty related to knee extension strength and very low certainty regarding BMD. Moderate to high confounding risk, small sample sizes along with large 95% CI, and distinct assessments were reasons for these findings.

## 4 Discussion

This systematic review and meta‐analysis investigated the effects of nandrolone decanoate supplementation on LST, fat mass, handgrip strength, knee extension strength and BMD in adults across various RCTs.

The most consistent finding was a statistically significant increase in LST following nandrolone decanoate supplementation compared to placebo, with a pooled MD of 1.59 kg. This aligns with previous work suggesting that anabolic steroids can enhance lean mass, likely through increased muscle protein synthesis and androgen receptor–mediated activation of satellite cells [67, 68]. However, this effect should be interpreted cautiously, as LST does not directly equate to functional muscle mass and may reflect fluid or non‐contractile tissue. More importantly, these gains were not accompanied by improvements in strength or BMD, which raises questions about their clinical relevance.

Within the included RCTs, adverse events associated with nandrolone decanoate were predominantly mild to moderate and reflected expected androgenic or administration‐related effects, including voice hoarseness, facial hair, fluid retention, injection‐site reactions and transient changes in blood pressure, haemoglobin or lipid profile [52, 53, 54, 57, 61, 66]. Several trials reported no clinically meaningful changes in renal function, liver enzymes, lipid profile or haematological parameters during the intervention period [48, 51, 59, 62, 63]. Serious adverse events were reported in a small number of studies, typically within trials enrolling older adults or clinical populations with substantial baseline health risk, and were not consistently attributed to nandrolone decanoate by the original trial authors [49, 64]. Importantly, most trials were short to medium in duration and not designed or powered to assess long‐term safety outcomes; therefore, concerns regarding cardiovascular, hepatic or endocrine risk are largely inferred from external literature rather than directly demonstrated within the present evidence base. Taken together, the dissociation between increased LST and the absence of consistent improvements in strength or BMD highlights the need for caution when inferring clinical benefit from these outcomes alone.

From a clinical perspective, the most relevant safety considerations relate to sex‐specific endocrine effects rather than cardiometabolic toxicity. In men, exogenous nandrolone decanoate suppresses the hypothalamic–pituitary–gonadal axis through negative feedback, leading to reduced endogenous testosterone and oestradiol production, with a transient hypogonadal state following cessation and associated reversible infertility [17, 69]. These effects are particularly pertinent given the musculoskeletal outcomes targeted in this review, as suppression of oestradiol may adversely affect bone turnover and BMD [70]. In women, prolonged exposure is associated with virilising effects, including voice deepening, hirsutism, clitoromegaly and menstrual disturbance, which may be irreversible and represent a major limitation to clinical use [17, 54].

Against this background, interpretation of anabolic effects remains limited by the sensitivity of commonly used outcome measures. This is particularly relevant given recent evidence from a systematic review by Aldrich et al., which highlighted the rapid onset and clinical impact of acute sarcopenia in hospitalised adults [71]. Their findings also highlight the limitations of conventional outcome measures such as LST or grip strength, which may underestimate meaningful physiological change. Advanced imaging‐based assessments of muscle quality (e.g., CT, MRI and ultrasound) may provide greater sensitivity in detecting clinically relevant muscle loss or recovery and should be prioritised in future trials. The observed increase in LST is consistent with other studies showing that nandrolone decanoate has a reliable anabolic effect, particularly when combined with resistance exercise or when administered in individuals with muscle wasting conditions such as those with HIV or undergoing haemodialysis [36, 38, 58]. Nonetheless, given that improvements in LST were not paralleled by changes in strength or BMD, the clinical utility of this anabolic response remains limited.

Our findings pertaining to grip strength changes found no significant increases, and evidence for knee extension strength was limited to a single trial, which reported no differences versus placebo. This suggests that increases in LST, may not translate into improvements in muscle strength and function—a key consideration in patient‐centred care. The disconnect between muscle quantity and muscle quality highlights the need to prioritise interventions that yield functional gains. This dissociation between structural and functional outcomes may be particularly relevant in clinical practice, where the aim is to primarily restore mobility and quality of life.

In contrast to LST, no significant changes in fat mass were observed. This suggests that although nandrolone decanoate may potentially promote muscle hypertrophy, it does not significantly affect body fat loss. However, it remains unclear whether a potential shift in fat distribution occurs, especially in more adipose‐prone regions, which may require more specific imaging techniques to assess [72]. Additionally, it is important to consider the long‐term cumulative effects of fat mass changes alongside improvements in LST. Existing studies lack sufficient long‐term exposure and follow‐up to assess chronic changes in fat mass following lean muscle gains with nandrolone decanoate. Hence, it would be important for future studies to consistently assess and report energy and protein intake to contextualise body composition outcomes.

Regarding muscle strength and functional outcomes, the effect of nandrolone decanoate on handgrip strength was not statistically significant, with a SMD of 0.39. Although there was a positive trend in handgrip strength improvement, the results were inconclusive, as reflected by the relatively wide confidence intervals. Further studies examining the relationship between nandrolone decanoate and handgrip strength, particularly those focused on older adults, are needed to clarify the potential benefits in improving upper body strength. Similarly, the effect of nandrolone decanoate on knee extension strength did not yield significant improvements. This may be due to the specific nature of knee extension exercises, which may not be as sensitive to the effects of anabolic steroids compared to exercises targeting larger muscle groups, such as squats or deadlifts. Another possibility is that the supplementation duration of 12 weeks may not have been long enough to induce significant improvements in strength, particularly in older adults or those with significant muscle loss. Nevertheless, the consistent lack of strength improvement suggests that LST gains alone are not a reliable proxy for functional recovery.

Effects on BMD were also inconclusive. No significant changes were observed in lumbar spine, radial or femoral neck BMD across studies, with most *p* values exceeding 0.05, suggesting that the supplementation did not have a significant impact. Although there is some evidence suggesting that nandrolone decanoate may have positive effects on BMD in specific populations, such as those with osteoporosis or osteopenia [54], its generalisability in the broader population remains limited. Reduced aromatisation of nandrolone compared with testosterone may also limit oestrogen‐mediated skeletal effects, which could partly explain the inconsistent BMD findings observed in men [73]. Given the lack of consistency and the absence of clinically meaningful changes, these findings do not currently support the use of nandrolone decanoate for bone health in general populations.

Despite the observed increase in LST, the potential risks of nandrolone decanoate cannot be overlooked. Whereas cardiometabolic adverse effects appear inconsistent or minimal in short‐term RCTs, sex‐specific endocrine consequences remain the primary safety‐limiting factor. Given that no consistent improvements were found in strength or BMD and that lean mass gains alone may not translate into clinical benefit, the case for nandrolone as a therapeutic agent is weak. Alternative short‐term strategies such as neuromuscular electrical stimulation or bespoke rehabilitation protocols may offer safer and more practical means of attenuating muscle loss during acute illness or hospitalisation [74, 75]. Taken together, the limited benefit and clear disadvantages suggest that the routine use of nandrolone decanoate for muscle maintenance should be approached with extreme caution, if not avoided altogether.

### 4.1 Strengths and Limitations

This is the first study to examine the effect of nandrolone decanoate supplementation on LST, fat mass, muscle strength and BMD compared with placebo. Our study employed multiple subgroup analyses to investigate different tissue compartments of BMD. However, our study was limited to a lack of research in terms of muscle strength, whereas simultaneously, the absence of dietary control weakens our ability to interpret changes primarily observed in LST and fat mass. This is critical to consider, given that different energy and/or protein intakes could have influenced our results. In addition, sex‐stratified analyses were not feasible due to inconsistent sex‐stratified outcome reporting and the under‐representation of female participants across included trials; however, sex was explored as a potential effect modifier using study‐level female proportion in an exploratory meta‐regression, which does not permit sex‐specific inference. Additionally, the relatively short‐exposure and follow‐up period provides limited long‐term data to understand the long‐term real‐world effects of nandrolone decanoate supplementation. Moreover, although study design and comparator groups were broadly similar, the included trials differed substantially in participant populations and health status, and several were conducted decades ago using outcome assessment methods, reporting standards and clinical protocols that are no longer routinely used. Pre‐specified physical performance outcomes (e.g., 6‐min walk test, SPPB, gait speed and timed up and go) were not reported in eligible placebo‐controlled trials and therefore could not be synthesised. Finally, six studies had some concerns in terms of risk of bias assessment primarily due to the lack of information on whether group allocation was concealed. Although *I* ^2^ values were low or 0% for two of our primary outcomes, this may reflect either genuine consistency across studies or limited statistical power to detect heterogeneity, particularly given the relatively small number of included trials.

## 5 Conclusions

In conclusion, nandrolone decanoate supplementation may lead to modest increases in LST but does not significantly affect fat mass, handgrip strength or knee extension strength in the short term. Its effect on BMD was inconsistent, with no significant improvements observed across various BMD sites. Taken together, the limited functional outcomes and known risks suggest that nandrolone decanoate offers little clinical value and should not be considered a routine intervention. In select, short‐term clinical scenarios, its use may be explored cautiously, but only under strict monitoring and in the absence of safer alternatives. Future research should prioritise safe, non‐pharmacological strategies—as resistance exercise, nutritional support and rehabilitation—as the default approach to maintain and restore musculoskeletal health.

## Funding

The authors have nothing to report.

## Ethics Statement

The authors of this manuscript certify that they comply with the ethical guidelines for authorship and publishing in the *Journal of Cachexia, Sarcopenia and Muscle* [76].

## Conflicts of Interest

The authors declare no conflicts of interest.

## Supporting information


**Data S1:** Supporting Information.

**Table S1:** Search terms employed in the screening of the literature search.

**Table S2:** Publication bias of the included studies.

**Table S3:** Meta‐regression analyses of the included studies.

**Table S4:** Risk of bias assessment of the included studies.

**Table S5:** GRADE assessment.

## Data Availability

Data are available upon request.
