# Supplementary material for: Effects of Nandrolone Decanoate on Muscle Strength, Body Composition and Bone Density: A Systematic Review and Meta‐Analysis
Source: J Cachexia Sarcopenia Muscle. 2026 Apr 5;17(2):e70276. doi: 10.1002/jcsm.70276 (PMC13052333; doi:10.1002/jcsm.70276)
Supplement: Supplementary file 1 — Data S1: Supporting Information. [file JCSM-17-e70276-s001.docx]

**Supplementary File.** Reported adverse events in each study related to nandrolone decanoate administration.

| **Study** | **Reported Adverse events** |
| --- | --- |
| Anstey 2022 | None in the monitored blood parameters (renal, liver function, and lipids) |
| Crawford 2003 | No significant differences between groups; Serious adverse events included: myocardial ischemia (n = 4), lung cancer (n = 2), pulmonary embolism (n = 2), ruptured aortic aneurysm (n = 2), carotid artery thrombosis (n = 1), cardio myopathy (n = 2), spinal stenosis (n = 1), and avascular necrosis of head of femur (n = 1) |
| Creutzberg 2003 | Lower erythrocyte sedimentation rate and increased lactate dehydrogenase vs. placebo |
| Daga 2014 | None in lipid profile, liver function tests, and blood pressure |
| Fahey 1973 | Transient water retention (n = 1); skin rash (n = 1); severe local reaction at the injection site (n = 1) |
| Flicker 1997 | 7% increase in systolic and 10% increase in diastolic blood pressure; 15% increase in plasma creatinine and aspartate aminotransferase; 6% increase in haemoglobin. One patient withdrew due to erythematous vocal cords; 4 withdrew due to leg oedema, 5 due to voice hoarseness (moderate cases), 5 due to facial hair (mild cases), and one due to inability to tolerate intramuscular injections because of anticoagulation. |
| Frisoli 2005 | Voice hoarseness (n = 4); soft facial hirsutism (n = 2) |
| Gold 2006 | Buttock pain on injection (n = 1); Ongoing weight increase (n = 1); Rash (n = 1); Non-specified pain (n = 1); Dehydration, diarrhoea, pyrexia and mouth ulceration (n = 1); respiratory insufficiency (n = 1) |
| Johansen 1989 | Did not report |
| Johansen 1999 | Hematoma at injection site (n = 1); Complaint about testicular size reduction (n = 1); Amenorrhea and acne (n = 2) |
| Johansen 2006 | Interference with sexual function (after five doses) and fear of possible adverse effects (after 3 doses) (n = unknown) |
| Lichtenbelt 2004 | Did not report |
| Lovejoy 1996 | Increased HDL and LDL cholesterol |
| Mulligan 2005 | No grade 3 or 4 elevations in transaminase or bilirubin levels, or total cholesterol |
| Passeri 1993 | Increased haemoglobin and decreased HDL cholesterol; No significant changes related to total cholesterol, triglycerides, creatinine, SGOT, SGPT, γ-GT, calcium, phosphate, parathyroid hormone, calcitonin, or somatodemin C; Increased facial hair, weight gain, increased blood pressure, hoarseness (n = 7). Weight gain was increased in 3 subjects (less than 2 kg), while blood pressure by 5-15 mmHg in 2 women |
| Sardar 2010 | No changes in haemoglobin, creatinine, liver enzymes, CD4+ count, and lipid profile |
| Sharma 2008 | No changes on blood pressure, complete blood count, serum glucose level, liver function tests, and lipid measurements |
| Sloan 1992 | Possible pulmonary embolus (n = 1); Became delirious within one week after surgery (n = 1) (hip fracture patient); Urinary obstruction requiring transurethral prostatectomy (n = 1); Depression (n = 1) |
| Storer 2005 | Peripheral oedema (n = 3); Increased haemoglobin and haematocrit; Decreased LH and FSH levels |
| Van Loan 1999 | Did not report |
