# Supplementary material for: Effects of Nandrolone Decanoate on Muscle Strength, Body Composition and Bone Density: A Systematic Review and Meta‐Analysis
Source: J Cachexia Sarcopenia Muscle. 2026 Apr 5;17(2):e70276. doi: 10.1002/jcsm.70276 (PMC13052333; doi:10.1002/jcsm.70276)
Supplement: Supplementary file 2 — Table S1: Search terms employed in the screening of the literature search. [file JCSM-17-e70276-s002.docx]

**Table S1.**Search terms employed in the screening of the literature search.

| **Database** | **Search terms** |
| --- | --- |
|  |  |
| PubMed | nandrolone AND ("*grip strength" OR "gait speed" OR "muscle size" OR "muscle mass" OR "appendicular lean"  OR "muscle loss" OR "muscle strength" OR frail* OR "physical performance" OR "physical capacity" OR "physical function"  OR "muscle power" OR "muscle force" OR "muscle function" OR "appendicular muscle mass" OR "lean mass" OR "fat free mass"  OR "chair stand test" OR "chair rise test" OR "sit-stand-test" OR "knee extension" OR "knee flexion" OR "quadriceps strength" OR  "lower limb strength" OR "stair climb" OR "timed up and go" OR "SPPB” OR “short physical performance battery” OR  "minute walk" OR "bone mineral density") |
| Cochrane Library | nandrolone AND ("*grip strength" OR "gait speed" OR "muscle size" OR "muscle mass" OR "appendicular lean"  OR "muscle loss" OR "muscle strength" OR frail* OR "physical performance" OR "physical capacity" OR "physical function"  OR "muscle power" OR "muscle force" OR "muscle function" OR "appendicular muscle mass" OR "lean mass" OR "fat free mass"  OR "chair stand test" OR "chair rise test" OR "sit-stand-test" OR "knee extension" OR "knee flexion" OR "quadriceps strength" OR  "lower limb strength" OR "stair climb" OR "timed up and go" OR "SPPB” OR “short physical performance battery” OR  "minute walk" OR "bone mineral density") |
| Web of Science | nandrolone AND ("grip strength" OR "gait speed" OR "muscle size" OR "muscle mass" OR "appendicular lean"  OR "muscle loss" OR "muscle strength" OR frail* OR "physical performance" OR "physical capacity" OR "physical function"  OR "muscle power" OR "muscle force" OR "muscle function" OR "appendicular muscle mass" OR "lean mass" OR "fat free mass"  OR "chair stand test" OR "chair rise test" OR "sit-stand-test" OR "knee extension" OR "knee flexion" OR "quadriceps strength" OR  "lower limb strength" OR "stair climb" OR "timed up and go" OR "SPPB” OR “short physical performance battery” OR  "minute walk" OR "bone mineral density") |
| Scopus | nandrolone AND ("*grip strength" OR "gait speed" OR "muscle size" OR "muscle mass" OR "appendicular lean"  OR "muscle loss" OR "muscle strength" OR frail* OR "physical performance" OR "physical capacity" OR "physical function"  OR "muscle power" OR "muscle force" OR "muscle function" OR "appendicular muscle mass" OR "lean mass" OR "fat free mass"  OR "chair stand test" OR "chair rise test" OR "sit-stand-test" OR "knee extension" OR "knee flexion" OR "quadriceps strength" OR  "lower limb strength" OR "stair climb" OR "timed up and go" OR "SPPB” OR “short physical performance battery” OR  "minute walk" OR "bone mineral density") |
