# Supplementary material for: Effects of Nandrolone Decanoate on Muscle Strength, Body Composition and Bone Density: A Systematic Review and Meta‐Analysis
Source: J Cachexia Sarcopenia Muscle. 2026 Apr 5;17(2):e70276. doi: 10.1002/jcsm.70276 (PMC13052333; doi:10.1002/jcsm.70276)

**Table S2.** Publication bias of the included studies.

Handgrip Strength


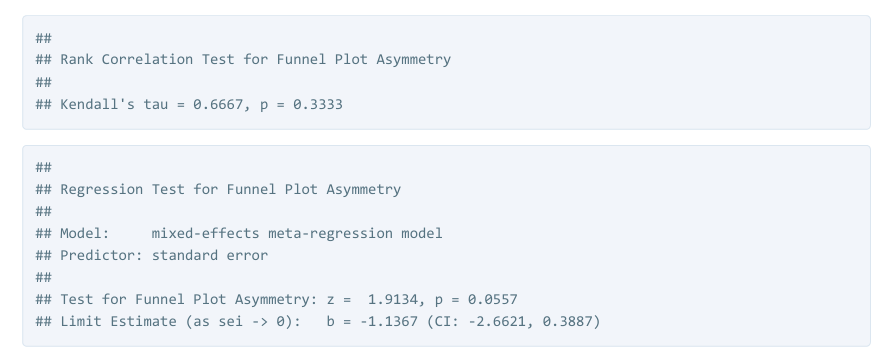


Funnel plot – Handgrip Strength


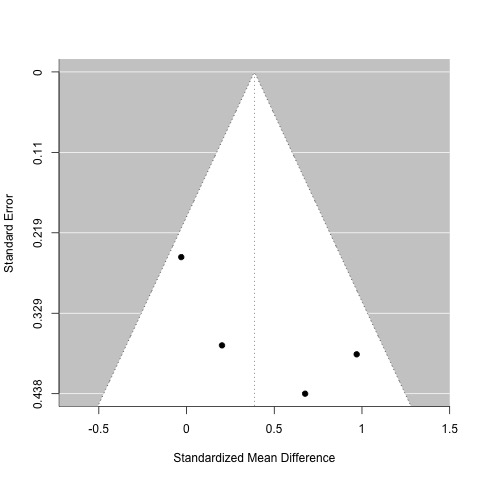


Lean Soft Tissue
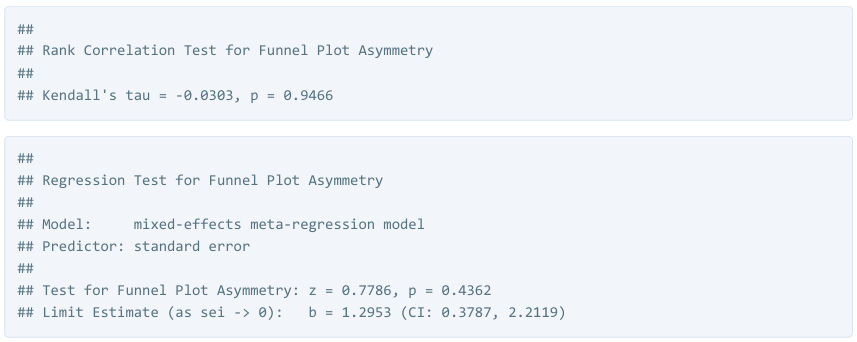


Funnel plot – Lean Soft Tissue


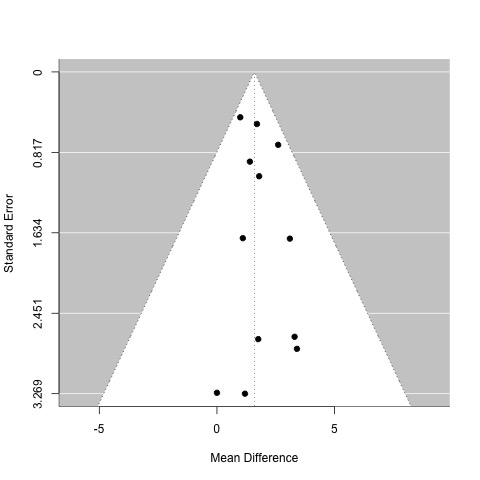


Fat Mass
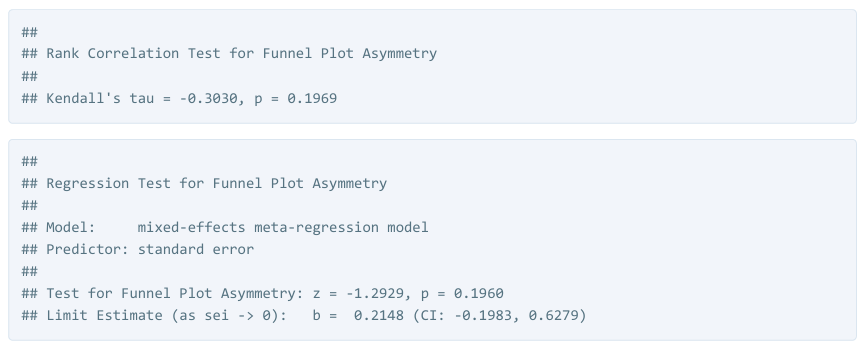


Funnel plot – Fat Mass


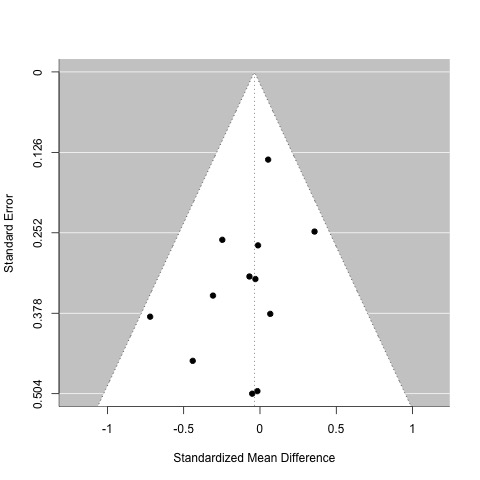

Supplement: Supplementary file 3 — Table S2: Publication bias of the included studies. [file JCSM-17-e70276-s006.docx]
