# Supplementary material for: Effects of Nandrolone Decanoate on Muscle Strength, Body Composition and Bone Density: A Systematic Review and Meta‐Analysis
Source: J Cachexia Sarcopenia Muscle. 2026 Apr 5;17(2):e70276. doi: 10.1002/jcsm.70276 (PMC13052333; doi:10.1002/jcsm.70276)
Supplement: Supplementary file 4 — Table S3: Meta‐regression analyses of the included studies. [file JCSM-17-e70276-s004.docx]

**Table S3.** Meta-regression analyses of the included studies.

**Handgrip Strength**


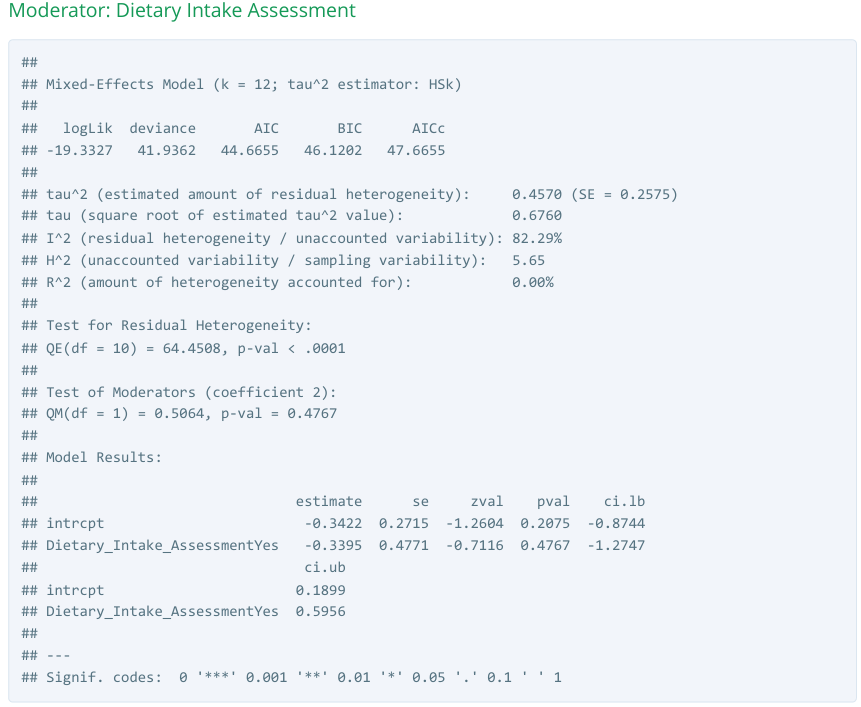


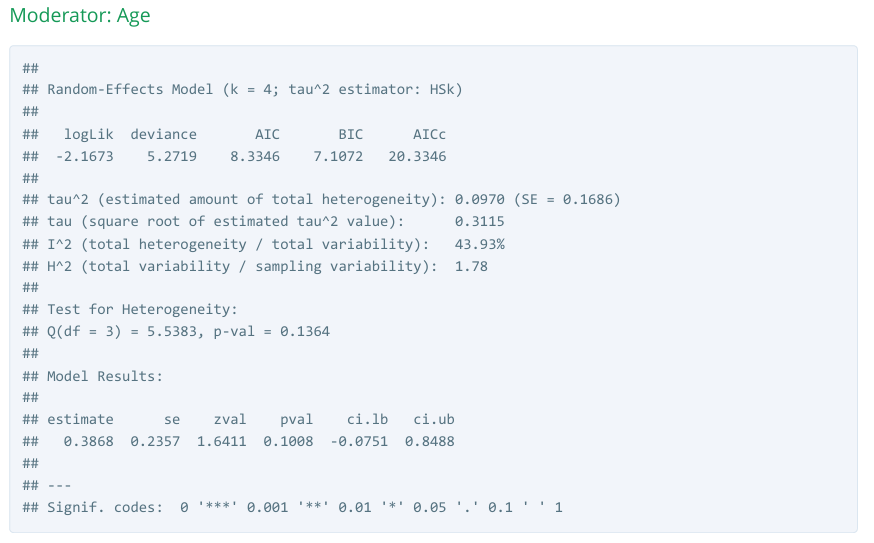

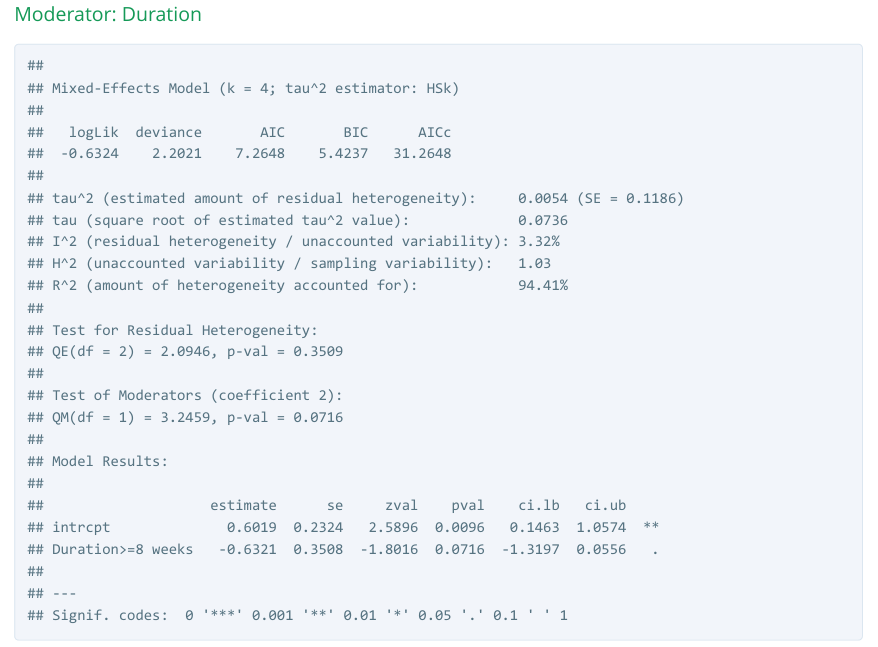


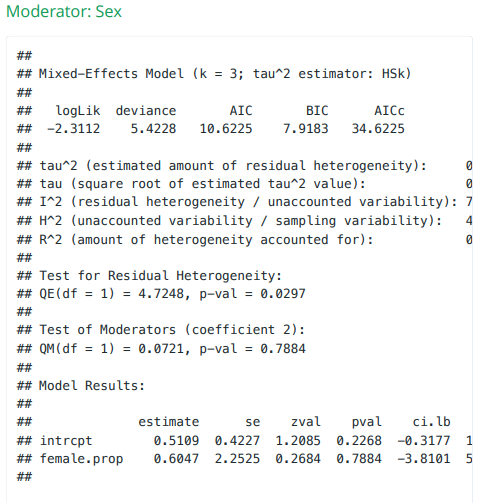


**Lean Soft Tissue**

**
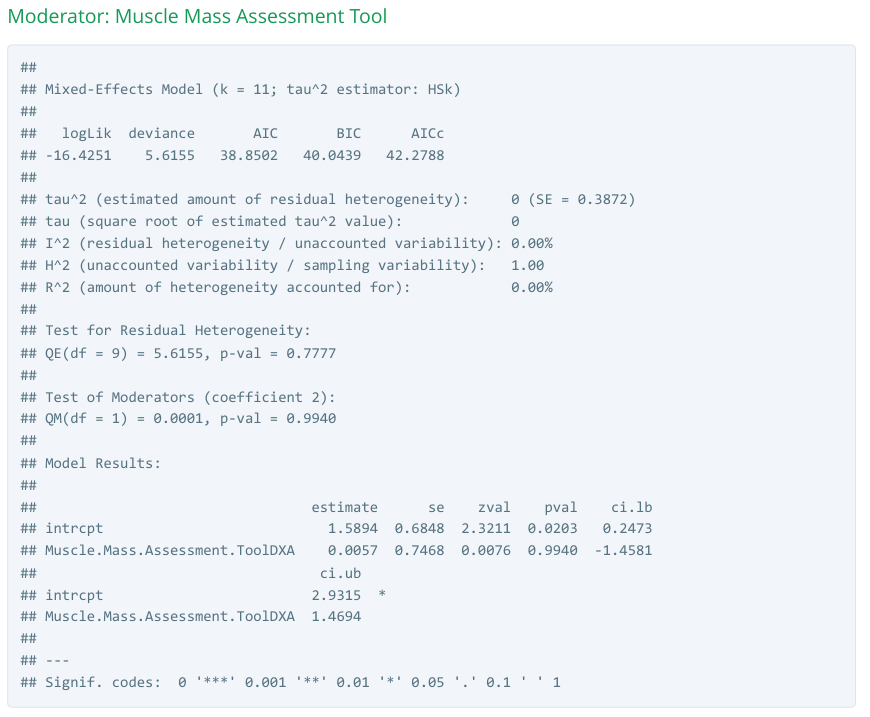
**

**
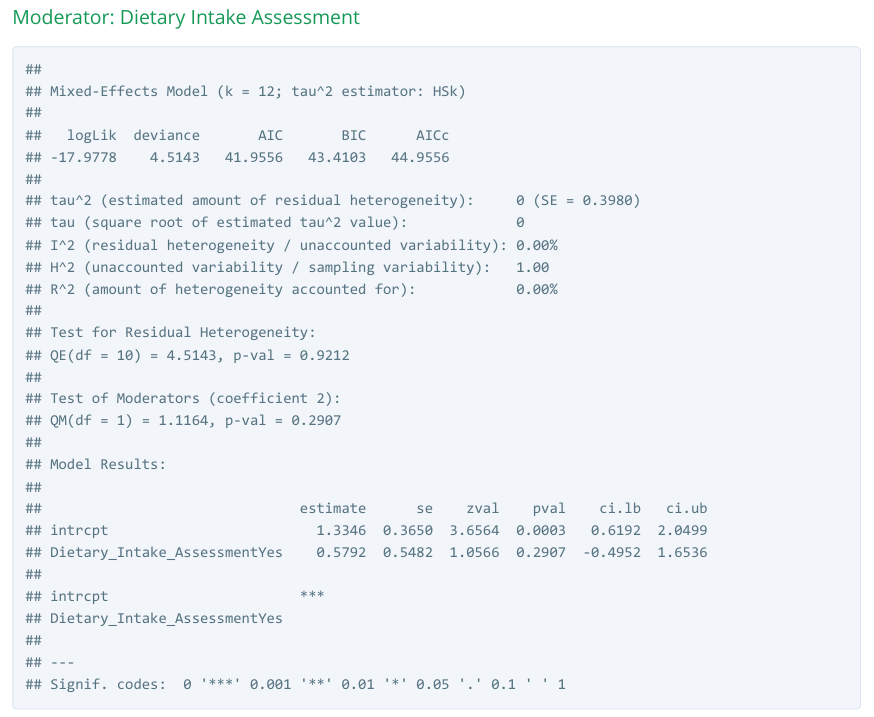
**

**
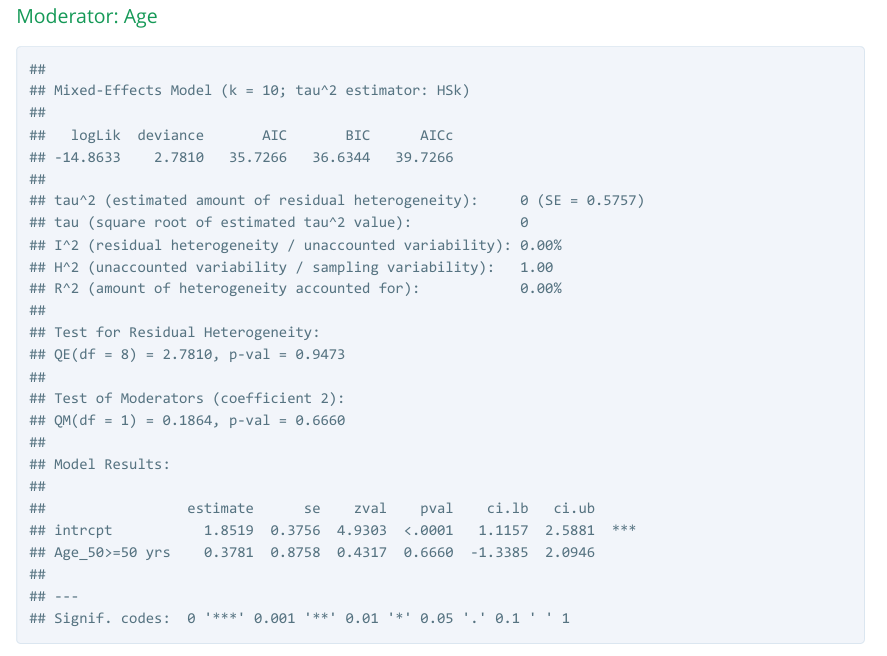
**

**
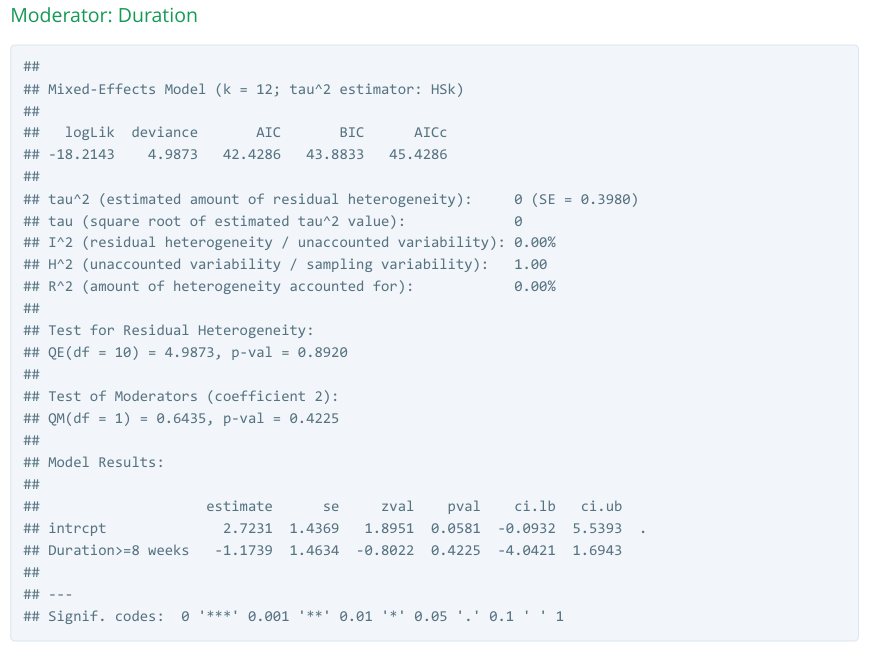
**

**
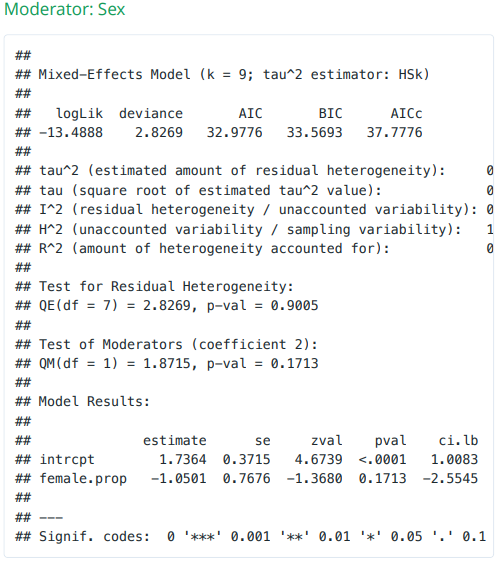
**

**Fat Mass**

**
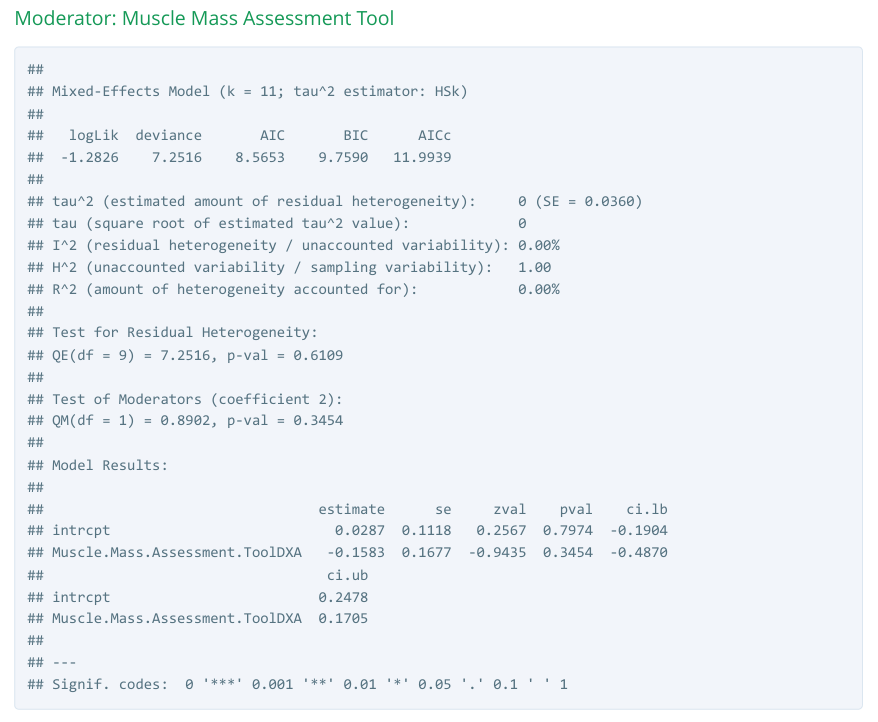
**

**
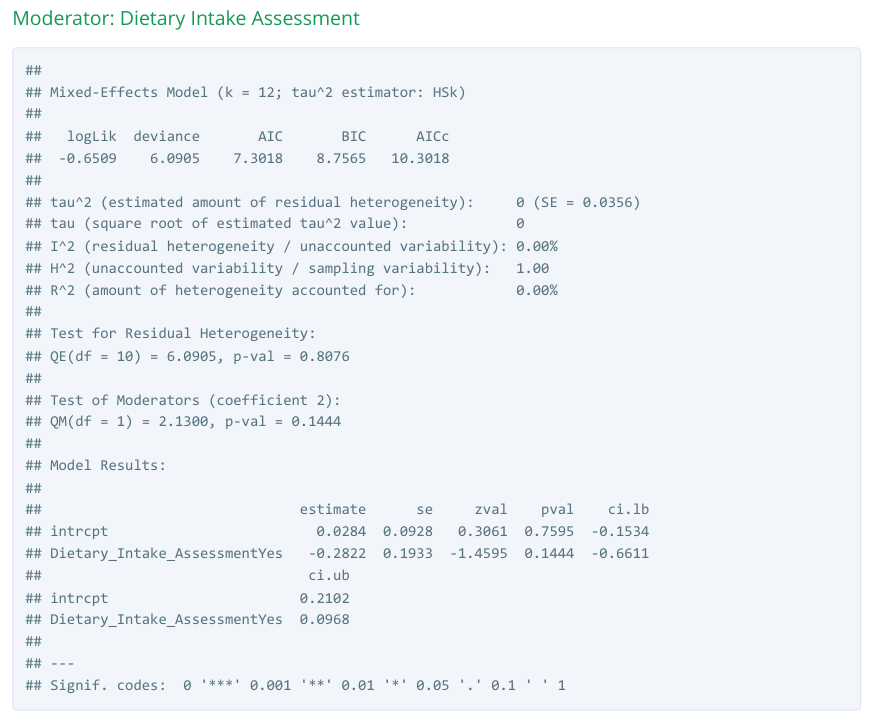
**

**
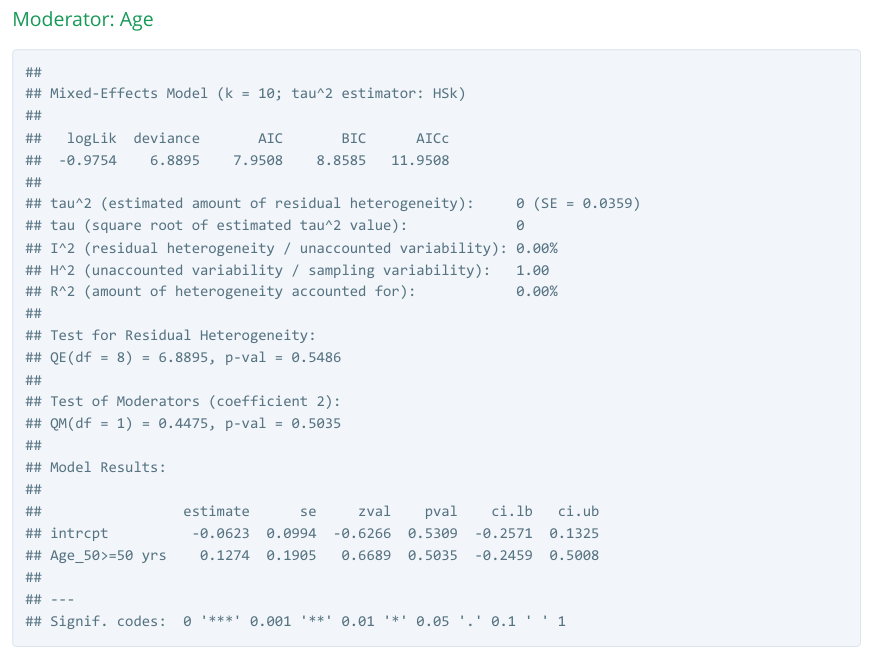
**

**
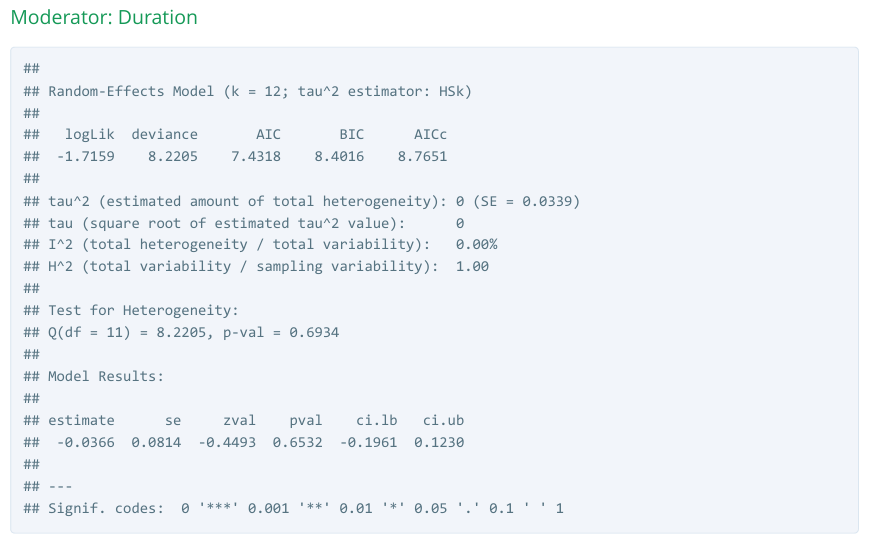
**

**
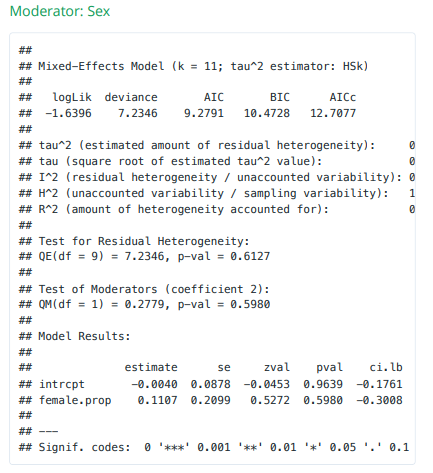
**
