# Supplementary figures and images for: Effects of Nandrolone Decanoate on Muscle Strength, Body Composition and Bone Density: A Systematic Review and Meta‐Analysis
Source: J Cachexia Sarcopenia Muscle. 2026 Apr 5;17(2):e70276. doi: 10.1002/jcsm.70276 (PMC13052333; doi:10.1002/jcsm.70276)

**Table S4.** Risk of bias assessment of the included studies.


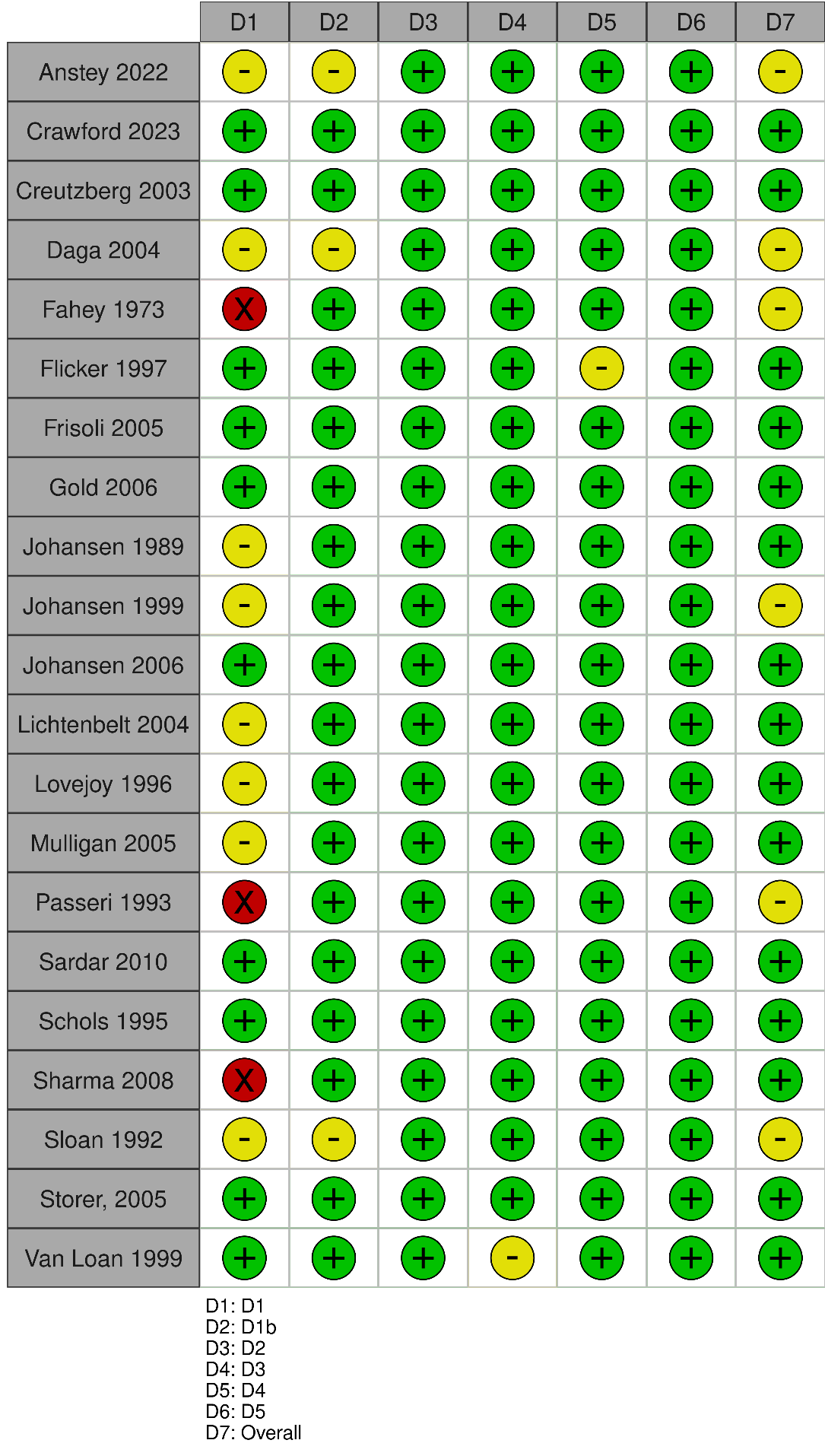

Supplement: Supplementary file 5 — Table S4: Risk of bias assessment of the included studies. [file JCSM-17-e70276-s003.docx]
