# Supplementary material for: Effects of Nandrolone Decanoate on Muscle Strength, Body Composition and Bone Density: A Systematic Review and Meta‐Analysis
Source: J Cachexia Sarcopenia Muscle. 2026 Apr 5;17(2):e70276. doi: 10.1002/jcsm.70276 (PMC13052333; doi:10.1002/jcsm.70276)
Supplement: Supplementary file 6 — Table S5: GRADE assessment. [file JCSM-17-e70276-s005.docx]

#### Table S5. GRADE assessment.

| Outcome № of participants (studies) | **Anticipated absolute effects (95% CI)** | Certainty | What happens |
| --- | --- | --- | --- |
|  | **Difference** |  |  |
| Fat mass change № of studies: (12 RCTs) | SMD **0.04 SD higher** (0.2 lower to 0.12 higher) | ⨁⨁◯◯ Low^a,b^ | Nandrolone may result in little to no difference in fat mass change. |
| Lean soft tissue change № of studies: (12 RCTs) | MD **1.59 higher** (1.06 higher to 2.13 higher) | ⨁⨁◯◯ Low^a,b^ | Nandrolone may increase lean soft tissue mass slightly. |
| Handgrip Strength change № of studies: (4 RCTs) | SMD **0.39 SD higher** (0.22 higher to 1.72 higher) | ⨁⨁◯◯ Low^a,b^ | Nandrolone may increase handgrip strength slightly. |
| Knee extension strength № of studies: (1 RCT) | SMD **0.29 SD higher** (0.15 lower to 0.73 higher) | ⨁⨁⨁◯ Moderate^b^ | Nandrolone likely results in little to no difference in knee extension. |
| Bone mineral density № of studies: (4 RCTs) | not pooled | ⨁◯◯◯ Very low^a,b,c^ | The evidence is very uncertain about the effect of nandrolone on bone mineral density. |

#### Explanations

a. Moderate to high confounding risk

b. Small sample size/large confidence intervals

c. Distinct local of assessment
